# A case report of hypokalemic periodic muscular weakness secondary to Sjögren's syndrome with distal renal tubular acidosis

**DOI:** 10.1002/ccr3.7769

**Published:** 2023-08-11

**Authors:** Marhaba Iqbal, Qaisar Ali Khan, Naod F. Belay, Moheem Azeem, Faiza Amatul‐Hadi, Muhammad Afzal, Harshawardhan Pande, Syed Yasir Shah, Rahma Ahmed, Sumaira Iram, Ravina Verma

**Affiliations:** ^1^ Khyber Teaching Hospital MTI KTH Peshawar Pakistan; ^2^ Michigan State University East Lansing Michigan USA; ^3^ Aga Khan University Karachi Pakistan; ^4^ Mercer University School of Medicine Macon Georgia USA; ^5^ St. George's University True Blue Grenada; ^6^ Saint Louis University St. Louis Missouri USA; ^7^ Kennesaw State University Kennesaw Georgia USA; ^8^ Sultan Qaboos University Muscat Oman

**Keywords:** hypokalemia, muscular weakness, renal tubular acidosis, Sjögren's syndrome

## Abstract

**Key Clinical Message:**

An underlying autoimmune condition should be suspected in patients who presented with periodic muscular weakness secondary to distal RTA that leads to hypokalemia because distal RTA is commonly associated with autoimmune disorders such as Sjögren's syndrome.

**Abstract:**

A 22‐year‐old female presented with a sudden onset of bilateral weakness in both upper and lower limbs. The patient had a history of muscular weakness secondary to hypokalemia and dryness of the eyes for the last 3 years. Laboratory investigations revealed decreased potassium and metabolic acidosis. Further investigations confirmed distal renal tubular acidosis (RTA) and Sjögren's syndrome. A diagnosis of distal RTA secondary to Sjögren's syndrome was made. Her potassium levels were replaced, and she was discharged with oral potassium supplements, steroids, and artificial tears.

## 1 INTRODUCTION

Sjögren's syndrome (SS) is a rare autoimmune condition typically characterized by chronic inflammation of exocrine glands such as the lacrimal salivary, resulting in symptoms like dryness of eyes and mouth. Extra‐glandular involvement in SS also occurs and includes glomerulonephritis, tubulointerstitial nephritis, arthritis, arthralgia, vasculitis, pulmonary diseases, and lymphomas.^1^ In the United States, the SS is thought to be the second most common rheumatological disease behind systemic lupus erythematosus (SLE), and female‐to‐male ratio is 9:1. The condition can affect an individual of any age but is more common in elderly population. Renal, pulmonary, and hematological involvements are more common among the Asian population.^2^, ^3^ Renal involvement is a well‐recognized extra‐glandular manifestation of primary SS, occurring in 16%–67% of cases, with distal renal tubular acidosis (RTA) being common, reported in 4.3%–9% of PSS patients.^4^

Renal tubular acidosis (RTA) is characterized by renal tubular impairment in maintaining physiological acid–base balance. It often results from defects in tubular transporters that participate in the secretion or uptake of specific ions. RTA can be caused by congenital factors, exposure to nephrotoxic drugs, diuretic abuse, autoimmune diseases, or malignancies. There are three major types of RTA: distal or type 1, proximal or type 2, and hyperkalemic or type 4. All three types of RTA are characterized by a positive urine anion gap, hyperchloremic nonanion gap metabolic acidosis, alkalotic or acidotic urine pH, and derangements in serum potassium levels (hypo‐ or hyperkalemia).^5^

We herein reported a case of distal RTA secondary to SS that led to hypokalemic periodic muscular weakness. This case report will enhance our understanding of renal involvement in patients with SS. Early identification and initiation of treatment for SS are crucial to reducing the risk of severe and potentially fatal complications. Additionally, clinicians should be aware of the potential renal involvements in these patients to provide timely and effective treatment and will provide evidence of the occurrence of renal tubular acidosis in SS patients.

## 2 CASE REPORT

A 22‐year‐old female patient who had a previous history of hypokalemia‐induced muscle weakness now presented to the emergency department of a tertiary care hospital with the complaint of increased muscle weakness in bilateral upper and lower limbs for 1 day. The weakness was painless and was not associated with a loss of consciousness. Previously, the patient was admitted for hypokalemia‐induced muscular weakness that was relieved after potassium replacement but her potassium level was not stable despite taking regular potassium supplements. She did not have any rash, joint pain, alopecia, and respiratory or gastrointestinal problems. There was no history of steroid use or laxative abuse. The weakness was prominent in both the proximal and distal extremities. Upon further inquiry, the patient reported dryness in the eyes for 3 years for which she was using artificial tears. Family history was nonsignificant for similar conditions. The patient was vitally stable and had a Glasgow coma score of 15/15. Thyroid examination was normal. She had no muscle tenderness, reflexes were 2/2, power was 3/5, the tone was 2/3 in both upper and lower limbs, sensations were intact, and plantar reflexes were down bilaterally. The Gower test was positive. Laboratory investigations revealed hypokalemia shown in Table 1.

**TABLE 1:** Lab investigations.

Laboratories	Values	Reference ranges
Hemoglobin	12.8	12.1–15.1 g/dL
WBC	9.05	4.5–11.0 × 109/L
Platelet	255	150–400 × 109/L
Sodium	138.7	135.0–150.0 mmol/L
Potassium	2.52	3.5–5.1 mmol/L
Chloride	115	96.0–112.0 mmol/L
Glucose	76.8	70–1140 mg/dL
Magnesium	2.4	1.5–2.5 mg/dL
Calcium	9.26	8–10 mg/dL
Total bilirubin	0.55	0.1–1.0 mg/dL
ALT/GPT	86.1	10–50 U/L
Alkaline phosphatase	132	35–104 U/L
Creatinine	0.86	0.42–1.06 mg/dL
PTH	8.88	7–53 pg/mL
TSH	2.15	0.3–4.2 mg/dL
Free T3	1.16	0.6–2.0 nmol/L
Free T4	15.04	10–28 pmol/L
HIV	Negative	‐
HBsAg	Negative	
HCV	Negative	‐

Abbreviations: ALT/GPT, alanine aminotransferase/glutamic‐pyruvic transaminase; HBsAg, hepatitis B surface antigen; hepatitis C virus; HIV, human immunodeficiency virus; mg/dL, milligram per deciliter; mmol/L, millimole per liter; nmol/L, nanomole per liter; pg/dL, picogram/deciliter; pmol/L, picomoles per liter; PTH, parathormone; TSH, thyroid‐stimulating hormone; U/L, units per liter; WBC, white blood cells.

Arterial blood gases of the patient were sent and showed normal anion gap metabolic acidosis with a pH of 7.21, bicarbonate of 16 mEq, and partial pressure of carbon dioxide (pCO_2_) of 36.5 mm Hg. All the other causes of hypokalemia with normal anion gap metabolic acidosis were ruled out by the patient history and laboratory investigations. A suspicious distal RTA that leads to hypokalemia was made, which was confirmed by urinary tests as shown in Table 2.

**TABLE 2:** Urinary electrolytes and anion gap.

Urine electrolytes	Value	Range
pH‐urine	7.0	4.5–7.8
Sodium	23	40‐220 mEq/L
Potassium	21.7	25‐125 mEq/L
Chloride	26	110‐250 mEq/L
Anion gap	18.7	3‐16 mEq/L

NH4Cl challenge test was not done due to the nonavailability of the test in our setup but ammonium excretion was indirectly measured by the urine anion gap. Based on the strong association of SS with distal RTA and dryness of the patient eyes, she was further investigated for SS, and her anti‐Sm/RNP antibodies, anti‐SSA/Ro antibodies, and anti‐SSB/La antibodies were positive. Additionally, a Schirmer test was done in which the patient scored less than 5 mm in 5 min. A final diagnosis of Sjögren's syndrome that led to distal RTA was made based on the American College of Rheumatology/European League Against Rheumatism (ACR‐EULAR) classification criteria for primary Sjögren's syndrome, in which the patient scored 4 out of 9.

Initially, the patient was given 2 ampules of injection KCL 150 mg/mL in 100 mL normal saline and infusion sodium bicarbonate 40 mEq. Her potassium level was raised to 3.1 mEq, and she was discharged home with a tablet of potassium citrate 2mMol/kg body weight, artificial tears, and skin lotion. After 4 weeks of follow‐up, the patient's potassium level remained stable at 4.0 mEq and she remained symptoms free for 3 months.

## 3 DISCUSSION

Renal involvement in primary SS has a prevalence of around 9%, although the first clinical manifestation with renal involvement is uncommon. Renal involvement in primary SS is either due to tubulointerstitial involvement or, less commonly, glomerular involvement. Lymphocytic infiltration of the kidney tubules by T cells, B cells, or plasma cells has been hypothesized to be behind the pathogenesis of renal involvement. Other causes include decreased hydrogen ion secretion secondary to the absence of a vacuolar H+ ATPase pump in the distal tubules and antibodies to the thiazide‐sensitive NaCl co‐transporter (NCCT). The presence of anti‐SSA, anti‐SSB, and hypergammaglobulinemia is the only risk factor predisposing to renal involvement in primary SS.^6^ All segments of the nephron can be involved in primary SS. This leads to distal and proximal RTA, Fanconi syndrome, diabetes insipidus, and, less commonly, Gitelman's syndrome and Bartter's syndrome. Of these, distal RTA is the most frequent tubular dysfunction in primary SS. Clinical features include mild hypokalemia, diabetes insipidus, renal tubular acidosis, profound hypokalemic periodic muscular weakness, and Fanconi syndrome.^7^ Glomerular involvement manifesting as proteinuria, hematuria, and severe renal dysfunction is uncommon. Renal tubular acidosis with severe hypokalemia causing muscular weakness is the most common manifestation of renal involvement in Sjögren's syndrome, although it is underdiagnosed.^7^, ^8^

In both primary and secondary Sjögren's syndrome, dRTA seems to be more prevalent in autoimmune diseases. Other autoimmune diseases such as systemic lupus erythematosus (SLE), primary biliary cirrhosis (PBC), autoimmune hepatitis (AIH), and rheumatoid arthritis are less commonly associated with dRTA.^9^ The association of dRTA with autoimmune diseases is listed in Table 3.

**TABLE 3:** Association of distal RTA with autoimmune diseases.

Authors	Patient	Age	Diagnosis	Presentation	Blood pH	Urine pH	Lab findings	Treatment	Outcome
Abdulla et al. (2017)^10^	Female	57	Sjögren's syndrome	SICCA, fracture, hypocalcemia, anemia (9.8 g/dL)	Ns	6.5	Serum K = 2.3 mmol/L HCO3 = 13 mmol/L Creatinine = 1.1 mg/dL	K+, HCO3‐, supplements HCQ	K+ correction
Ammad Ud Din et al. (2020)^11^	Female	44	SLE	Hypokalemic paralysis, SICCA, proteinuria	7.21	6.5	Serum K = 1.6 mmol/L HCO3 = 10 mmol/L Creatinine = 0.8 mg/dL	K+, HCO3− supplements; prednisone 20 → 40 mg/day, HCQ	K+ correction, proteinuria remission
Elitok et al. (2020)^12^	Female	60	Primary biliary cirrhosis	Progressive chronic kidney disease, leukocyturia	7.23	7	Serum K = 3.3 mmol/L HCO3 = 11.1 mmol/L Creatinine = 1.93 mg/dL	Not specified	Not specified
Duarte Silveira et al. (2022)^13^	Female	67	Rheumatoid arthritis	Peripheral arterial disease, hypomagnesemia	7.38	6.5	Serum K = 3.1 mmol/L HCO3 = 15.7 mmol/L Creatinine = 0.51 mg/dL	K+ supplements	Not specified

The latest and new American College of Rheumatology/European League Against Rheumatism (ACR‐EULAR) classification criteria for primary Sjögren's syndrome are the result of international collaboration and have been derived using a well‐established and validated methodology. Classification of primary Sjögren's syndrome is assigned to anyone who meets the inclusion criteria and scores four out of five when the weights from the five criteria in the table are combined. A score of 4 was achieved after positive results from the anti‐SSA and anti‐Ro tests, along with Schirmer's test. A positive anti‐SSA/anti‐Ro antibody, a positive Schirmer's test, dry eyes, and mouth, as well as a positive minor salivary gland biopsy, are all criteria in Table 4. All five criteria of primary Sjögren's syndrome were met in the patient, so the weights from each criterion were added up to give the patient a score of 4.

**TABLE 4:** American College of Rheumatology/European League Against Rheumatism (ACR‐EULAR) classification criteria for primary Sjögren's syndrome.^14^

Items	Weight/Score
Labial salivary gland with focal lymphocytic sialadenitis and focus score of ≥1 foci/4 mm2	3
Anti‐SSA/anti‐Ro positive	3
Ocular staining score ≥5 (or van Bijsterveld score ≥4) in at least one eye	1
Schirmer's test ≤5 mm/5 min in at least one eye	1
Unstimulated whole saliva flow rate ≤0.1 mL min−1	1

Treatment of Sjögren's syndrome depends on initial symptoms and extra‐glandular manifestations. Severe and acute systemic manifestations require the treatment of corticosteroids with or without immunosuppressive agents. Management of distal RTA is essentially supportive, including potassium and bicarbonate supplementations, and close follow‐up to prevent complications from nephrolithiasis. Corticosteroids are used to reduce inflammation and modulate the immune response, while immunosuppressive agents help further reduce the activity of the immune system. Potassium and bicarbonate supplementation helps maintain electrolyte balance and prevent the development of nephrolithiasis, which can cause further complications. Early diagnosis and lifelong alkali supplementation can prevent both acute hypokalemia and chronic complications like osteomalacia, renal stones, and progression to chronic kidney disease.^15^ Our patient responded well to treatment with prednisone. By supplementing potassium and bicarbonate, the kidneys can maintain electrolyte balance, which prevents the formation of nephrolithiasis. Additionally, prednisone helps reduce symptoms associated with the condition, such as inflammation and swelling and improves the patient's overall well‐being. As curative treatments remain to be elucidated, monitoring for complications such as nephrocalcinosis, nephrolithiasis, and rickets/osteomalacia should also take place along with ongoing treatment of metabolic derangements and symptoms.^16^

## 4 CONCLUSION

Distal RTA sometimes presents clinically with the sign and symptoms of hypokalemia such as muscular weakness, and the condition is often secondary to other systemic diseases such as SLE and Sjögren's syndrome. Early detection and prompt management of primary etiology are necessary for a better outcome. Treatment of distal RTA mainly involves supportive care and electrolyte replacement.

## AUTHOR CONTRIBUTIONS

**Marhaba Iqbal:** Conceptualization; data curation. **Qaisar Ali Khan:** Data curation; investigation; methodology; writing – review and editing. **Naod F. Belay:** Writing – original draft. **Moheem Azeem:** Conceptualization; data curation. **Faiza Amatul‐Hadi:** Writing – review and editing. **Muhammad Afzal:** Writing – original draft. **Harshawardhan Pande:** Writing – original draft. **Syed Yasir Shah:** Writing – original draft. **Rahma Ahmed:** Writing – original draft. **Sumaira Iram:** Methodology; validation. **Ravina Verma:** Formal analysis; project administration; supervision.

## FUNDING INFORMATION

The authors received no funding for this research.

## CONFLICT OF INTEREST STATEMENT

The authors declare no conflict of interest.

## ETHICS STATEMENT

Not required.

## CONSENT STATEMENT

Written informed consent was obtained from the patient to publish this case report. Consent can be provided upon the Editors‐in‐chief request.

## Data Availability

Data can be available upon reasonable request to the corresponding author.
